# DELIRIUM AND CARDIOVASCULAR DISEASE IN OLDER ADULTS WITH COVID-19

**DOI:** 10.1093/geroni/igae098.3945

**Published:** 2024-12-31

**Authors:** Esther Park, Milenko Petrovic, Gohar Azhar

**Affiliations:** University of Arkansas for Medical Sciences, Little Rock, Arkansas, United States; University of Arkansas for Medical Sciences, Little Rock, Arkansas, United States; University of Arkansas for Medical Sciences, Little Rock, Arkansas, United States

## Abstract

SARS-COV-2 is often associated with cardiovascular diseases such as arrhythmias and congestive cardiac failure (CHF), and cardiovascular disease is itself linked with the development of delirium. In this retrospective study we explored the relationship between heart disease and acute delirium in 319 COVID-19 patients 65 and older, seen at the University of Arkansas for Medical Sciences from January 7, 2018 to January 5, 2021. We reveiwed the frequency of cardiac conditions (atrial fibrillation, atrial flutter, tachycardia, bradycardia, CHF, CAD, other) in this cohort with emphasis on timing before and concurrent with COVID-19 delirium. We found a strong association of acute delirium during COVID-19 infection in patients with cardiac disease as compared to patients without cardiac disease (p< 0.00001). 128/319 (40.1%) COVID-19 patients suffered from delirium during or after COVID-19, and 88 of these patients had cardiac disease. 121/319 (37.9%) COVID-19 patients had no other cardiac conditions excluding essential hypertension. The majority (83/121, 68.6%) of those with no cardiac conditions listed in EMR also had no delirium documented during or after infection. Patients with cardiac conditions had a risk ratio of 1.38 of developing delirium during or after COVID-19 infection compared to patients without COVID-19. In conclusion, having a preexisting cardiac condition increases the likelihood of delirium in older adults with COVID-19 infection. Improved treatment strategies that optimize cardiovascular care during COVID-19 will also benefit cognition and enhance outcomes in older adults. A better understanding of these complex interactions should improve treatment of delirium in older adults with SARS-COV-2.

